# LSTM-conformal forecasting-based bitcoin forecasting method for enhancing reliability

**DOI:** 10.1371/journal.pone.0319008

**Published:** 2025-05-02

**Authors:** Xiangyue Zhang, Yuyun Kang, Chao Li, Wenjing Wang, Keqing Wang

**Affiliations:** 1 School of Information Science and Engineering, Linyi University, Linyi, Shandong, China; 2 School of Logistics, Linyi University, Linyi, Shandong, China; 3 Daopuyun (Shandong) Intelligent Technology Co., Ltd., Jinan, Shandong, China; Beijing Technology and Business University, CHINA

## Abstract

Cryptocurrency is a new type of asset that has emerged with the advancement of financial technology, creating significant opportunities for research. bitcoin is the most valuable cryptocurrency and holds significant research value. However, due to the significant fluctuations in bitcoin’s value in recent years, predicting its value and ensuring the reliability of these predictions, which have become crucial, have gained increasing importance. A method that combines Long Short-term Memory (LSTM) with conformal prediction is proposed in this paper. Initially, the high-dimensional features in the dataset are divided using the Spearman correlation coefficient method, and features below 0.75 and above 0.95 are excluded. Subsequently, an LSTM model is built, and data are fed into it and the data is used to train the model to generate predictions. Finally, the predicted values generated by the LSTM are fed into the conformal prediction model, and confidence intervals for these values are generated to verify their reliability. In the conformal prediction model, the quantile loss of the loss function is defined, and an Average Coverage Interval (ACI) predictor is designed to improve the accuracy of the results. The experiments are conducted using data from CoinGecko, which is a publicly available data. The results show that the LSTM-conformal prediction (LSTM-CP) combination improves reliability.

## 1. Introduction

**Fig 1:**
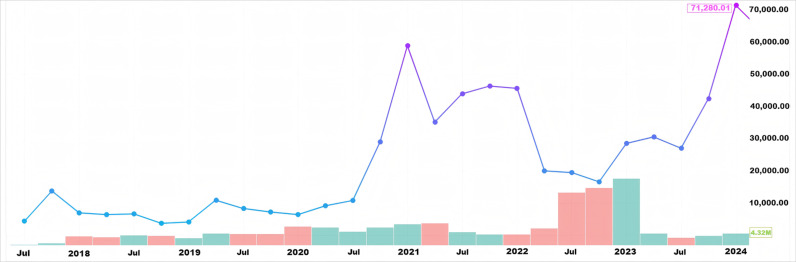
The trends in bitcoin prices from 2018 to 2024.

Bitcoin value prediction is crucial for market traders. Recently, bitcoin has rapidly dominated the trading market. Recently, turmoil in the international financial markets has led to significant fluctuations in bitcoin market transactions (as shown in Fig 1). Accurate bitcoin value predictions can lead to significant returns for investors and businesses. For instance, accurate predictions enable individual investors to make informed decisions. Predicting future price movements allows investors to optimize returns or minimize losses by deciding whether to buy, hold, or sell. Financial analysts and businesses can analyze market trends and devise strategies using bitcoin value predictions. For example, if forecasts show a rising bitcoin value, businesses might increase their bitcoin holdings or offer bitcoin payments. Additionally, value forecasting serves as a vital risk management tool. Understanding potential price swings enables investors and businesses to plan and protect against extreme market fluctuations. This involves strategies such as stop-loss orders and portfolio diversification.

In the early days, scholars extensively researched bitcoin value prediction. Sin et al. [1] introduced a selective neural network ensemble method based on genetic algorithms for predicting bitcoin value. The relationship between bitcoin price changes over the next day and its features is explored, using a multilayer perceptron for each feature in an ensemble neural network. Experimental results showed that this strategy generated nearly 85% returns, outperforming the “previous day trend following” strategy (38% return) and the best single Multi-layer Perceptron (MLP) model strategy (53% return). Afterward, Struga et al. [2] adopted an LSTM-based recurrent neural network for bitcoin price prediction, demonstrating the effectiveness of the LSTM architecture on time series data. Results included bitcoin price predictions for the next 30 and 60 days. Meanwhile, Azari et al. [3] demonstrated the effectiveness of the Autoregressive Integrated Moving Average (ARIMA) model in predicting the value of bitcoin over three years. Empirical studies found it effective in stable periods, especially for short-term, one-day forecasts. However, given bitcoin’s price volatility, training ARIMA for more than three years or long-term forecasts would introduce significant errors.

On this basis, more in-depth research is conducted on previous studies. Poongodi et al. [4] explored the technology of the bitcoin network and diverse machine learning prediction algorithms. The public bitcoin blockchain data set (April 28, 2013 - July 31, 2017) is used [5], and ARIMA is applied for price prediction. Afterward, A deep learning method, the Stacked Denoising Autoencoder (SDAE), is used to predict bitcoin prices by Liu et al. [6]. The results showed that the SDAE model outperformed popular machine learning methods like Back-propagation Neural Network (BPNN) and Support Vector Regression (SVR) in both directional and horizontal predictions. To investigate the dynamic tail risk of the bitcoin market. Gao et al. [7] use the Conditional Autoregressive Value-at-risk (CAViaR) model to jointly estimate Value-at-risk (VaR) and Expected Shortfall (ES) to explore the dynamic tail risk of the bitcoin market. To achieve more accurate measurements, we constructed a Markov Regime-switching (MS) model with time-varying transition probabilities driven by information from asset price bubbles. Experimental results using daily bitcoin data from 2013 to 2021 provide strong evidence for structural changes in bitcoin’s VaR and ES. Furthermore, the bubble index significantly impacts tail risk and enhances the model’s ability to estimate and predict VaR and ES. In the subsequent study, Chen et al. [8] combined random forest regression and LSTM to create a highly accurate model for next-day bitcoin price prediction, detailing an impact analysis showing that the model with a single lagged explanatory variable had a negative impact on next-day bitcoin price prediction. The currency price prediction had the highest accuracy. Moreover, Ranjan et al. [9] adopted a variety of machine learning techniques to predict bitcoin prices. Experimental results showed that logistic regression could predict daily price predictions with 64.84% accuracy, while eXtreme Gradient Boostin (XGBoost) could predict 5-minute intervals with 59.4% accuracy. Following this, Koo et al. [10] introduced Concentrated Cluster Distribution (CCD) as a new data filtering method to improve bitcoin price prediction by resolving its extreme bimodality. This improved tail and overall prediction accuracy. Experimental results showed that with LSTM and SSA decomposition, the CCD-WES strategy surpassed the baseline by reducing the Root Mean Square Error (RMSE) by 11.5% and 22.5% in the overall and extreme cases, respectively.

Although the number of bitcoin value prediction methods is increasing and their accuracy is improving, studies on reliability remain scarce. We emphasize that predictions should not only be accurate but also reliable. Therefore, A method combining LSTM with conformal prediction is proposed in this article to enhance prediction reliability.

The design of LSTM, which is tailored for time series data processing and forecasting, offers unique advantages in predicting bitcoin’s value. LSTM’s gating mechanism allows it to capture long-term dependencies. It enables LSTM to “remember” key past data for future predictions. Moreover, LSTM’s structure offers robustness against data outliers and noise. Most notably, it exhibits significant dynamic adaptability. As market conditions and behaviors evolve, so do stock conditions. LSTM continuously learns from new data, adapting to changes and ensuring predictions remain accurate and reliable. This guarantees LSTM’s peak performance in predicting bitcoin values.

Conformal forecasting provides a unique approach to predicting bitcoin’s value by quantifying uncertainty and enhancing decision-making confidence. Its primary advantage lies in providing confidence intervals for predictions, thereby effectively quantifying uncertainty. It offers not just a point estimate of bitcoin’s expected price but also a range that could encompass the actual price. Moreover, confidence intervals allow bitcoin users, both individuals and businesses, to conduct more accurate risk assessments. For instance, a wider confidence interval suggests higher volatility, indicating a prudent holding strategy. Importantly, the conformal prediction framework applies to various models (such as LSTM, linear regression, random forests), offering a universal method for quantifying uncertainty.

The structure of this paper is presented as follows. The common bitcoin prediction model, LSTM, ACI predictor and conformal prediction are introduced in section 2: Related work. The evaluation metrics of the conformal prediction model are detailed in section 3: Evaluation criteria. The dataset used, and the processing and partitioning of high-dimensional features in the dataset using the Spearman correlation coefficient method are described in section 4: Dataset. The experimental results, including advantages and disadvantages, are discussed in section 5: Experiment analysis. The main findings are concluded in section 6: Conclusion.

## 2. Related work

### 2.1. Bitcoin value forecasting

#### 2.1.1. Methods in bitcoin value forecasting.

**Fig 2:**
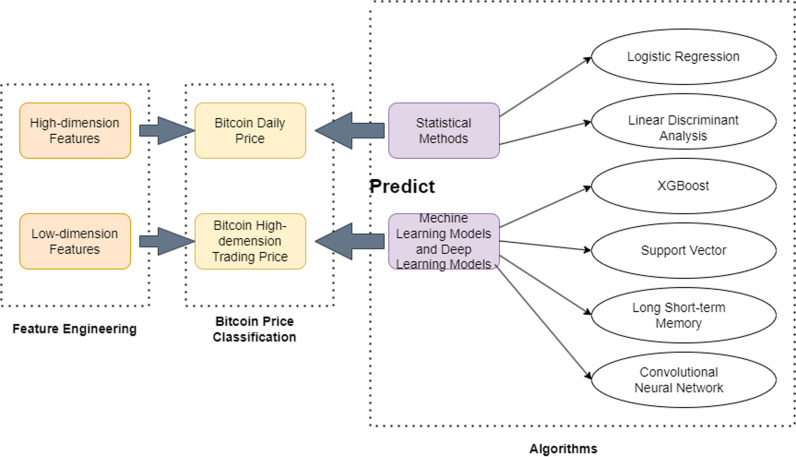
An overview of the framework used by machine learning methods for bitcoin price prediction.

With the continuous development of artificial intelligence, bitcoin predictions have expanded beyond traditional statistics and mathematics. Currently, bitcoin prediction methods tend to combine both approaches. Consequently, machine learning methods like XGBoost and Support Vector Machines (SVM), as well as deep learning methods like long short-term memory networks and Convolutional Neural Network (CNN), demonstrate better performance. An overview of the research framework is shown in Fig 2.

#### 2.1.2. Classical bitcoin forecasting methods.

Traditional methods for predicting bitcoin prices typically involve gathering data from social media, news reports, market comments, and other channels. Sentiment analysis seeks to comprehend how the market reacts to specific news events and their potential impact on price movements. A method for predicting cryptocurrency prices using news and historical price data is described by Vo et al. [11]. The findings indicate that sentiment analysis plays a key role in predicting cryptocurrency prices, given the interactive nature of financial activities. In the subsequent study, the use of sentiment analysis as a computational tool to predict the price of bitcoin and other cryptocurrencies over different time intervals is proposed by Wolk et al. [12]. Twitter and Google trends are used to predict short-term prices of major cryptocurrencies, highlighting how these social media platforms influence buying decisions. The findings indicate that people’s psychological and behavioral attitudes significantly influence the prices of highly speculative cryptocurrencies.

#### 2.1.3. Machine learning methods for bitcoin forecasting.

The advent of machine learning has brought about innovative prediction methods for cryptocurrencies like bitcoin. bitcoin prices are predicted by McNally et al. [13] using Bayesian optimized Recurrent Neural Network (RNN) and LSTM networks. The results showed that the nonlinear deep learning method outperformed the less effective ARIMA prediction. The deep learning model is benchmarked on both GPU and CPU, and it trained 67.7% faster on GPU than on CPU. In the subsequent work, a framework for predicting daily bitcoin prices using advanced machine learning methods, taking into account specific exogenous and endogenous factors, is examined by Dutta et al. [14]. RMSE is used as the evaluation metric. Experimental results showed that the Gated Recurrent Unit (GRU) model with recurrent dropout outperformed other well-known models. Subsequently, Hafid et al. [15] proposed a classification machine learning method to predict the market direction, whether it will be up or down. Key features including the Relative Strength Index (RSI) and Moving Average Convergence Divergence (MACD) are identified, and the data are fed into a machine learning model. Evaluation results showed that the proposed machine learning method could generate buy and sell signals with an accuracy of over 86%.

#### 2.1.4. Deep learning methods for bitcoin forecasting.

The emergence of deep learning has introduced a wider range of bitcoin prediction methods. Efficient deep learning-based prediction models, specifically utilizing LSTM and GRU, are developed by Awoke et al. [16] to resolve bitcoin price fluctuations with high accuracy. Their study compared these two time series deep learning techniques and demonstrated their effectiveness in predicting bitcoin prices. In the subsequent study, Jiang et al. [17] explored various deep learning networks and methods to improve Precision, such as min-max normalization, Adam optimizer, and windowed min-max normalization. It is found that MLPs are not suitable for predicting prices based on current trends due to lack of memory. LSTM models, especially when combined with past memory and GRU, provided the most accurate predictions.

#### 2.1.5. Hybrid model for bitcoin forecasting.

Theoretical and empirical studies indicate that the integration of various models, particularly from different methodologies, enhances predictive performance. Hybrid models are based on the rationale that a single model might not adequately capture the data-generating process or encompass all characteristics of the time series. Hybrid models reduce the risk of failure and enhance outcomes by merging multiple models to decrease dependence on any single, potentially inappropriate model.

Numerous studies have explored hybrid forecasting methods, frequently merging traditional and artificial intelligence techniques. A novel hybrid model is proposed by Hashish et al. [18] to address the challenge from both a descriptive and predictive perspective. Hashish employs hidden Markov models to describe the historical trends of cryptocurrencies and long short-term memory networks to predict future trends. The model proposed by Hashish has proven to be effective compared to traditional time series forecasting models such as ARIMA and traditional LSTM. In the subsequent study, Gao et al. [19] proposed a hybrid method that combines the advantages of non-stationary parametric models, such as Generalized Autoregressive Conditional Heteroskedasticity (GARCH), with the nonlinear modeling capabilities of LSTM neural networks. The results show that their hybrid model achieves similar predictive performance in terms of Mean Square Error (MSE), Mean Absolute Error (MAE), and RMSE, but in terms of precision, accuracy, and F1-score with optimal hyperparameters.

### 2.2. LSTM

**Fig 3:**
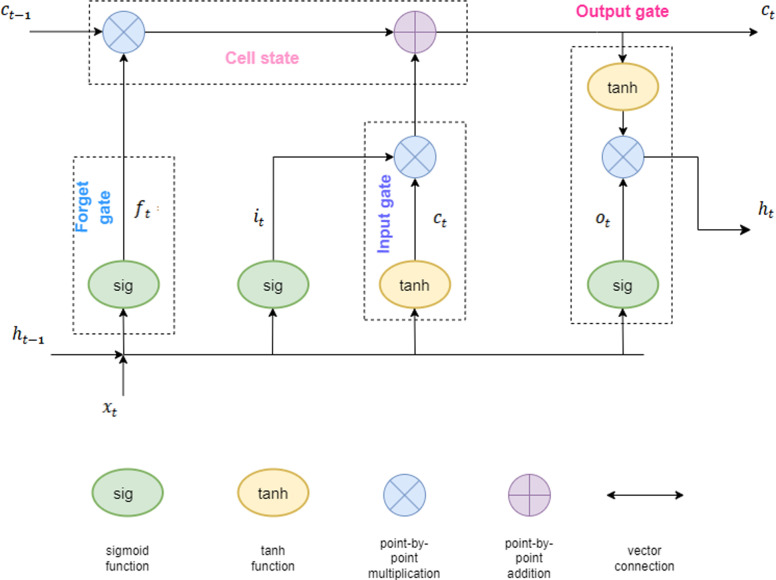
LSTM framework diagram.

As shown in Fig 3, LSTM is a special type of RNN designed to process and predict long-term dependencies in time series data. LSTM addresses the problems of gradient vanishing and explosion in traditional RNNs by introducing three gates: input gate, forget gate, and output gate. The input gate controls the reception of new information, the forget gate determines how much old information to retain, and the output gate controls the transition from cell state to the final output. This gate structure allows LSTM to retain past information and use it when necessary. First, it decides what information to discard through the forget gate. Second, the input gate decides which new information to store in the cell state. Then, the cell state is updated to incorporate new information. Finally, the output gate determines the output based on the cell state and the current input.

In the LSTM network, the equations for each time step operation are as follows, and Table 1 summarizes the notation used in this study:

**Table 1:** A summary of the symbols used in the equations shown in LSTM.

Notation	Definition
$f_{t}$	The forget gate $f_{t}$ determines how much of the previous cell state $c_{t-1}$ is retained.
$i_{t}$	The input gate $i_{t}$ decides how much new input information is stored in the cell state.
$o_{t}$	The output gate $o_{t}$ determines how much of the current cell state $c_{t}$ should be output.
$c_{t}$	The cell state $c_{t}$ is the core of the LSTM, preserving crucial information over time.
$h_{t}$	The hidden state $h_{t}$ contains the output information of the current time step.
*W* and *U*	W is the weight matrix for the input $x_{t}$, while *U* is the weight matrix for the previous time step’s hidden state ${\text{h}}_{\text{t}-1}$.
*b*	Bias vector.
$x_{t}$	Input at time *t*.
$h_{t-1}$	Hidden state of the previous time step.
$c_{t-1}$	Cell state of the previous time step.
*σ*	The Sigmoid activation function, used to control the gates.
tanh	The hyperbolic tangent activation function, used to process cell states and help regulate information flow.


$$f_{t}=\sigma \left(W_{f}x_{t}+U_{f}h_{t-1}+b_{f}\right),$$ (1)

$$i_{t}=\sigma \left(W_{t}x_{t}+U_{1}h_{t-1}+b_{t}\right),$$ (2)

$$o_{t}=\sigma \left(W_{o}x_{t}+U_{0}h_{t-1}+b_{o}\right),$$ (3)

$$c_{t}=\left(f\times c_{t-1}\right)+\left(i_{t}\times \sigma_{c}\right)\times \left(W_{c}x_{t}+U_{c}h_{t-1}+b_{c}\right),$$ (4)

$$h_{t}=o_{t}\times \text{tanh}\left(c_{t}\right).$$ (5)

### 2.3. Conformal prediction

Conformal prediction [20] is a statistical framework that generates uncertainty intervals for the prediction of machine learning models. This study reviews several distribution-free prediction methods that provide guarantees under the assumption of exchangeability of the data and symmetry of the underlying algorithm.

**Split conformal prediction:** Split conformal prediction [21], also known as inductive conformal prediction, is a holdout method used to construct prediction intervals around a pre-trained model. Specifically, given a model μ:X→R fitted on an initial training data set, and n additional data points $(X_{1},Y_{1}),...,\left((X_{n},Y_{n}\right)$ (the holdout set), residuals are defined.


$$R_{i}=\left|Y_{i}-\hat{\mu }\left(X_{i}\right)\right|,i=1,\ldots ,n,$$ (6)

and then compute the prediction interval at the new feature vector ${\text{X}}_{\text{n}+1}$ as


$${\hat{C}}_{n}\left(X_{n+1}\right)=\hat{\mu }\left(X_{n+1}\right)\pm \left(\text{the }�\text{(1-}\alpha )\left(n+1\right)�-\text{th smallest of }R_{1},\ldots ,R_{n}\right).$$ (7)

Equivalently,


$${\hat{C}}_{n}\left(X_{n+1}\right)=\hat{\mu }\left(X_{n+1}\right)\pm {\text{Q}}_{1-\alpha }\left(\overset{n}{\underset{i=1}{\sum }}\frac{1}{n+1}\cdot \delta_{R_{i}}+\frac{1}{n+1}\cdot \delta_{+\infty }\right).$$ (8)

where $\text{Qτ}\left(\cdot \right)$ denotes the τ -quantile of its argument, and δ a denotes the point mass at a.

This method guarantees distribution-free predictive coverage at the target level 1−α.

A drawback of the split conformal method is the loss of accuracy due to sample splitting, as the pretrained model μ must be independent of the holdout set. In practice, if only n labeled data points are available, n/2 points are used for training μ, and the procedure defined in equation (8) is run with a holdout set of size n/2 instead of n.

In this study, n will continue to denote the holdout set size for the split conformal method, to maintain consistent notation across different methods.

**Full conformal prediction:** An alternative to avoid the cost of data splitting is the full conformal method, also known as transductive conformal prediction.


$$A:\underset{n\geq 0}{�}{\left(X\times \mathbb{R}\right)}^{n}\to \left\{\text{measurable functions }\hat{\mu }:X\to \mathbb{R}\right\}.$$ (9)

Consider a regression algorithm: $\text{n}\geq 0\left(\text{X}\times \text{R}\right)\text{n}\to$ {measurable functions μ:X→R, which maps a dataset containing pairs ($X_{i},Y_{i}$) to a fitted regression function μ.The algorithm *A* must treat data points symmetrically, i.e.,


$$A\left(\left(x_{\pi \left(1\right)},y_{\pi \left(1\right)}\right),\ldots ,\left(x_{\pi \left(n\right)},y_{\pi \left(n\right)}\right)\right)=A\left(\left(x_{1},y_{1}\right),\ldots ,\left(x_{n},y_{n}\right)\right)$$ (10)

$\text{A}\left(\text{xπn}\left(1\right),\text{yπ}\left(1\right)\right),-\left(\text{xπ}\left(\text{n}\right),\text{yπ}\left(\text{n}\right)\right)=$$\text{A}\left(\text{x}1,\text{y}1\right),\ldots ,\left(\text{xn},\text{yn}\right)$ for all n≥1, all permutations π on $\left[\text{n}\right]:=\left\{1,\ldots ,\text{n}\right\}$, and all $\left\{\left(\text{xi},\text{yi}\right)\right\}\text{i}=1,\ldots ,\text{n}$. For each y∈R,


$${\hat{\mu }}^{y}=A\left(\left(X_{1},Y_{1}\right),\ldots ,\left(X_{n},Y_{n}\right),\left(X_{n+1},y\right)\right).$$ (11)

let $\text{μy}=\text{A}\left(\text{X}1,\text{Y}1\right)$, $\ldots ,\left(\text{Xn},\text{Yn}\right),\left(\text{Xn}+1,\text{y}\right)$ denote the trained model, fitted to the training data and a hypothesized test point (Xn+1,y), and let


$$R_{i}^{y}= \{\begin{array}{ll} \left|Y_{i}-{\hat{\mu }}^{y}\left(X_{i}\right)\right|, & i=1,\ldots ,n,\\ \left|y-{\hat{\mu }}^{y}\left(X_{n+1}\right)\right|, & i=n+1. \end{array}.$$ (12)

The prediction set (which may or may not be an interval) for feature vector Xn+1 is defined as


$${\hat{\text{C}}}_{\text{n}}\left({\text{X}}_{\text{n}+1}\right)=\left\{\text{y}\in \mathbb{R}:{\text{R}}_{\text{n}+1}^{\text{y}}\leq {\text{Q}}_{1-\text{α}}\left(\overset{\text{n}+1}{\underset{\text{i}=1}{\sum }}\frac{1}{\text{n}+1}\cdot {\text{δ}}_{{\text{R}}_{\text{i}}^{\text{y}}}\right)\right\}.$$ (13)

The full conformal method guarantees distribution-free predictive coverage at the target level 1−α:

**Theorem 1** (Full conformal prediction: If the data points (X1, Y1), ... , (Xn, Yn), (Xn + 1, Yn + 1) are i.i.d. (or more generally, exchangeable), and the algorithm *A* treats the input data points symmetrically as in (10), then the full conformal prediction set defined in (13) satisfies.


$$\mathbb{P}\left\{Y_{n+1}\in {\hat{C}}_{n}\left(X_{n+1}\right)\right\}\geq 1-\alpha .$$ (14)

The same result holds true for split conformal.

In this study, we explore conformal prediction methods, specifically variants of split conformal prediction, also known as inductive conformal prediction. Next, we delineate the principal procedure of split conformal prediction for a general input *x* and output *y*.

**Step 1.** Split a dataset into two complementary subsets, the training fold $D_{tr}$ and the calibration fold $D_{cal}$, with the calibration fold containing n samples.

**Step 2.** Use the training fold ${\text{D}}_{\text{tr}}$ to train a deep learning model.

**Step 3.** Define a non-conformity score function $s\left(a,y\right)$ that quantifies how atypical a pair $\left(a,y\right)$.

**Step 4.** Calculate the quantile $\hat{q}$ at the $\frac{\left(n+1\right)\left(1-\alpha \right)}{n}$ level for the non-conformity scores obtained from the calibration set $D_{cal}$.

**Step 5.** Use the quantile $\hat{q}$ to generate the uncertainty intervals for predictions on a new instance, $x_{test}$:


$$C\left(x_{\text{test }}\right)=\left\{y:s\left(x_{\text{test }},y\right)\leq \hat{q}\right\}$$ (15)

The prediction set always satisfies a validity property for any score function and data distribution under the i.i.d condition. The coverage guarantee is formally provided next.

**Theorem 2.** Conformal coverage guarantee [22]. Suppose ${\left({\text{x}}_{\text{i}},{\text{y}}_{\text{i}}\right)}_{\text{i}=1,\ldots ,\text{n}}$ and $\left({\text{x}}_{\text{test }},{\text{y}}_{\text{test }}\right)$ are i.i.d. and define $\hat{\text{q}}$ as in step 4 above and $C\left({\text{X}}_{\text{test }}\right)$ as in step 5 above. Then, the following statement holds.:


$$\mathbb{P}\left(y_{\text{test }}\in C\left(x_{\text{test }}\right)\right)\geq 1-\alpha$$ (16)

Meanwhile, Theorem 1 also holds on the assumption of exchangeability [23]. The i.i.d. assumption in the above Theorem is stronger than the assumption of exchangeability that is practicable in the real world, Table 2 summarizes the notation used in this paper.

**Table 2:** A summary of the symbols used in the equations shown in CP.

Notation	Definition
*n*	Size of the calibration set $D_{cal}$.
*α*	Confidence level parameter.
$C\left(x_{\text{test }}\right)$	Prediction set containing all candidate values *y* for the new instance $x_{\text{test }}$.
$s\left(x_{\text{test }},y\right)$	Non-conformity score for the new instance $x_{\text{test }}$ and candidate value *y*.
$y_{\text{test }}$	True value of the response variable for the new instance.
*i.i.d*	independent and identically distributed

In summary, the bitcoin market often exhibits extreme price fluctuations, which can render traditional time series forecasting methods (such as ARIMA or conventional machine learning models) ineffective in predicting future prices, especially during sudden market events or sentiment shifts. Conformal forecasting addresses this challenge effectively. Conformal forecasting provides both a point forecast (such as a single price value) and a forecast interval (confidence interval). This interval is dynamically generated based on the distribution characteristics of both training and new data, reflecting the uncertainty of the forecast. In a highly volatile market like bitcoin, the forecast interval shows the range of possible price fluctuations, providing decision makers with more comprehensive information. Conformal forecasting does not rely on assumptions about the distribution of data. Even with high volatility and changing statistical properties over time, conformal forecasting adapts to market changes by dynamically adjusting the forecast interval and reducing the model’s sensitivity to extreme fluctuations.

Conformal prediction effectively handles non-stationarity. A key feature of conformal prediction is its independence from the assumption of data stationarity. Therefore, (1) it can be applied to non-stationary time series to learn and generate predictions directly from raw data. Despite the bitcoin market’s highly non-stationary characteristics, conformal forecasting provides meaningful forecast intervals to aid decision-making. (2) The conformal forecasting method avoids the complex step of stabilizing the data through a “non-parametric” method. It adjusts to the data at each moment, adapts to the market’s non-stationary characteristics, and responds to new trends.

### 2.4. ACI predictor

In this study, the ACI predictor is selected. The ACI predictor operates on the principles of conformal prediction theory in statistics. Conformal prediction, a powerful statistical tool, guarantees a certain coverage probability for predictions without assuming any specific data distribution.

Suppose we are given a fitted regression model for predicting the value of *Y* from *X*. Consider *y* as a candidate value for $Y_{t}$. To assess if $\hat{y}$ is a reasonable estimate of $Y_{t}$, we define a conformity score $S\left(X,Y\right)$ to measure the extent to which y aligns with our model’s predictions. For instance, if our model yields point predictions $\hat{\mu }\left(X_{t}\right)$, a conformity score could evaluate the distance between $\hat{\mu }\left(X_{t}\right)$ and y. An example of this is:


$$S\left(X_{t},y\right)=\left|\hat{\mu }\left(X_{t}\right)-y\right|,$$ (17)

alternatively, let’s assume our regression model produces estimates, $\hat{Q}\left(X;p\right)$, for the $p^{th}$ quantile of the Y�X distribution. We could then apply Conformal Quantile Regression (CQR), a method that evaluates the signed distance between y and the estimated upper and lower quantiles via a score.


$$S\left(X_{t},y\right)=max\left\{\hat{Q}\left(X_{t};\alpha /2\right)-y,y-\hat{Q}\left(X_{t};1-\alpha /2\right)\right\}.$$ (18)

Regardless of the chosen conformity score, the primary challenge is determining the threshold for $S\left(X_{t},y\right)$ such that $\hat{y}$ is considered a reasonable prediction for $Y_{t}$. Assume we have a calibration set $D_{cal}$ = $\left\{\left(X_{r},Y_{r}\right)\right\}$$\begin{array}{c} t-1\\ r=1 \end{array}$ that is different from the data that is used to fit the regression model. Using this calibration set,the conformity scores’ fitted quantiles are defined as


$$\hat{q}\left(p\right)=inf\left\{s:\frac{1}{\left|D_{\text{cal }}\right|}\underset{\left(x,y\right)\in D_{\text{cal }}}{\sum }{�}_{s\left(x,y\right)\leq s}\geq p\right\}.$$ (19)

A reasonable prediction for $Y_{t}$ is *y* if $\left(X_{t},y\right)$$\leq \hat{q}\left(1-\alpha \right)$. The key observation is that if the data $D_{cal}\cup \left\{\left(X_{t},Y_{t}\right)\right\}$ are exchangeable and ties are broken uniformly at random, the rank of $S\left(X_{t},Y_{t}\right)$ among ${\left\{S\left(X_{n},Y_{n}\right)\right\}}_{\left(X_{n},Y_{n}\right)\in D_{\text{cal }}}\cup$$\left\{S\left(X_{t},Y_{t}\right)\right\}$ will follow a uniform distribution. Therefore,


$$P\left(S\left(X_{t},Y_{t}\right)\leq \hat{q}\left(1-\alpha \right)\right)=\frac{\left|D_{\text{cal }}\right|\left(1-\alpha \right)}{\left|D_{\text{cal }}\right|+1},$$ (20)

thus, defining our prediction set as


$${\hat{C}}_{t}=\left\{y:S\left(X_{t},y\right)\leq \hat{q}\left(1-\alpha \right)\right\},$$ (21)

provides a marginal coverage guarantee.


$$P\left(Y_{t}\in {\hat{C}}_{t}\right)=P\left(S\left(X_{t},Y_{t}\right)\leq \hat{q}\left(1-\alpha \right)\right)=\frac{\left|D_{\text{cal }}\right|\left(1-\alpha \right)}{\left|D_{\text{cal }}\right|+1}.$$ (22)

By introducing additional randomization this generic procedure can be altered slightly to produce ${\hat{C}}_{t}$ that satisfies the exact marginal coverage guarantee $P\left(Y_{t}\in {\hat{C}}_{t}\right)=1-\alpha$. Additionally, it is noteworthy that the described method is commonly known as split or inductive conformal inference. This refers to splitting the observed data into a training set for fitting the regression model and a separate calibration set, Table 3 summarizes the notation used in this paper.

**Table 3:** A summary of the symbols used in the equations shown in ACI.

Notation	Definition
$S\left(X_{t},y\right)$	Conformity score.
$X_{t}$	Predictor variable.
*y*	Candidate value for the response variable.
$\hat{\mu }\left(X_{t}\right)$	Model prediction value based on $X_{t}$ (point prediction of the regression model).
$\hat{Q}\left(X_{t};p\right)$	The $p^{th}$ quantile of the distribution of *Y* given $X_{t}$.
*α*	Significance level parameter, usually set to 0.05.
$\hat{q}\left(p\right)$	Combined quantile.
inf	Infimum.
*s*	Candidate value in the range.
$\left|D_{\text{cal }}\right|$	Size of the calibration set $\left|D_{\text{cal }}\right|$.
$\left(x,y\right)$	Data pair in the calibration set.
$\hat{q}\left(1-\alpha \right)$	Quantile of 1−α.

## 3. Evaluation criteria

This study employs the quantile loss function, RMSE, MAE and Mean Absolute Percentage Error (MAPE) as evaluation metrics. The quantile loss function is applied in quantile regression. Unlike traditional loss functions, the quantile loss function imposes varying penalties on predictions for low and high values, enabling the model to more effectively capture different aspects of the data distribution. The quantile loss function employs different τ values to mitigate overfitting and underfitting, thereby enabling effective quantile regression. y represents the true value of each observation. $\hat{\text{y}}$ denotes the predicted value of the model corresponding to each observation. Table 4 provides a summary of the symbols used in this paper.

**Table 4:** A summary of the symbols used in the equations shown in quantile loss function.

Notation	Definition
$J_{\text{quant }}$	Quantile loss, which is used to evaluate the model’s prediction error in quantile regression.
*N*	Total number of observations.
$y_{i}$	True value of the response variable for the $i^{th}$ observation.
*τ*	Quantile level, a parameter that determines the weight of the loss function.
${\hat{y}}_{i}\geq y_{i}$	The loss function is $\tau \cdot \left|y_{i}-{\hat{y}}_{i}\right|$, which means that when the model underestimates the true value, the loss is weighted by *τ*.
${\hat{y}}_{i}$ $y_{i}$	The loss function is $\left(1-\tau \right)\cdot \left|y_{i}-{\hat{y}}_{i}\right|$,which means that when the model overestimates the true value, the loss is weighted by 1−τ.


$${\text{J}}_{\text{quant }}=\frac{1}{N}\overset{N}{\underset{i=1}{\sum }}\left[\tau \left|y_{i}-{\hat{y}}_{i}\right|{�}_{y_{i}\geq {\hat{y}}_{i}}+\left(1-\tau \right)\left|y_{i}-{\hat{y}}_{i}\right|{�}_{y_{i}<{\hat{y}}_{i}}\right],$$ (23)

$$\text{MAE }=\frac{1}{n}\overset{n}{\underset{i=1}{\sum }}\left|y_{i}-{\hat{y}}_{i}\right|,$$ (24)

$$\text{RMSE }=\sqrt{\frac{1}{n}\overset{n}{\underset{i=1}{\sum }}{\left(y_{i}-{\hat{y}}_{i}\right)}^{2}},$$ (25)

$$\text{MAPE}=\frac{1}{n}\overset{n}{\underset{i=1}{\sum }}\left|\frac{y_{i}-{\hat{y}}_{i}}{y_{i}}\right|\times 100\%.$$ (26)

When ${\hat{\text{y}}}_{\text{i}}\geq {\text{y}}_{\text{i}}$ (the true value is greater than or equal to the predicted value), the loss function is τ· $\left({\text{y}}_{\text{i}}-{\hat{\text{y}}}_{\text{i}}\right)$. This implies that the loss is weighted by the value of τ if the model underestimates the true value. Where, τ serves as a weight that amplifies the error when predicted values are low. Correspondingly, when ${\hat{\text{y}}}_{\text{i}}$ < ${\text{y}}_{\text{i}}$ (the true value is smaller than the predicted value), the loss function is $\left(1-\text{τ}\right)$· (${\hat{\text{y}}}_{\text{i}}-{\text{y}}_{\text{i}}$), This implies that the loss is weighted by the value of $\left(1-\text{τ}\right)$ if the model overestimates the true value. Where, $\left(1-\text{τ}\right)$ serves as a weight that amplifies the error when predicted values are high.

## 4. Dataset

In this study, we used bitcoin prices from 2018 to 2024 (Fig 1). Additionally, we utilized bitcoin blockchain information and various statistics [5]. We considered 29 features of the bitcoin blockchain among various available features (as shown in Table 5).

**Table 5:** Bitcoin blockchain features.

Feature	Description
avg-block-size	The 24 h average block size in MB.
blockchain-size	The total size of all block headers and transactions.
cost-per-trans	Miners revenue divided by the number of transactions.
cost-per-trans-pct	Miners revenue as percentage of the transaction volume.
difficulty	A relative measure of difficulty in finding a new block.
est-trans-vol	The estimated value of transactions on the bitcoin blockchain in BTC.
est-trans-vol-usd	The estimated USD value of transactions.
hash-rate	The estimated number of tera hashes per second the bitcoin network is performing.
market-cap	The total USD value of bitcoin supply in circulation.
market-price	The average USD market price across major bitcoin exchanges.
med-cfm-time	The median time for a transaction to be accepted into a mined block.
mempool-count	The number of transactions waiting to be confirmed.
mempool-growth	The rate of the memory pool (mempool) growth per second.
mempool-size	The aggregate size of transactions waiting to be confirmed.
miners-revenue	The total value of Coinbase block rewards and transaction fees paid to miners.
my-wallets	The total number of blockchain wallets created.
n-trans	The number of daily confirmed bitcoin transactions.
n-trans-excl-100	The total number of transactions per day excluding the chains longer than 100.
n-trans-excl-popular	The total number of transactions, excluding those involving any of the network’s 100 most popular addresses.
n-trans-per-block	The average number of transactions per block.
n-trans-total	The total number of transactions.
n-unique-addr	The total number of unique addresses used on the bitcoin blockchain.
output-val	The total value of all transaction outputs per day.
total-bitcoins	The total number of bitcoins that have already been mined.
trade-vol	The total USD value of trading volume on major bitcoin exchanges.
trans-fees	The total BTC value of all transaction fees paid to miners.
trans-fees-usd	The total USD value of all transaction fees paid to miners.
trans-per-sec	The number of bitcoin transactions added to the mempool per second.
utxo-count	The number of unspent bitcoin transactions outputs.

**Fig 4:**
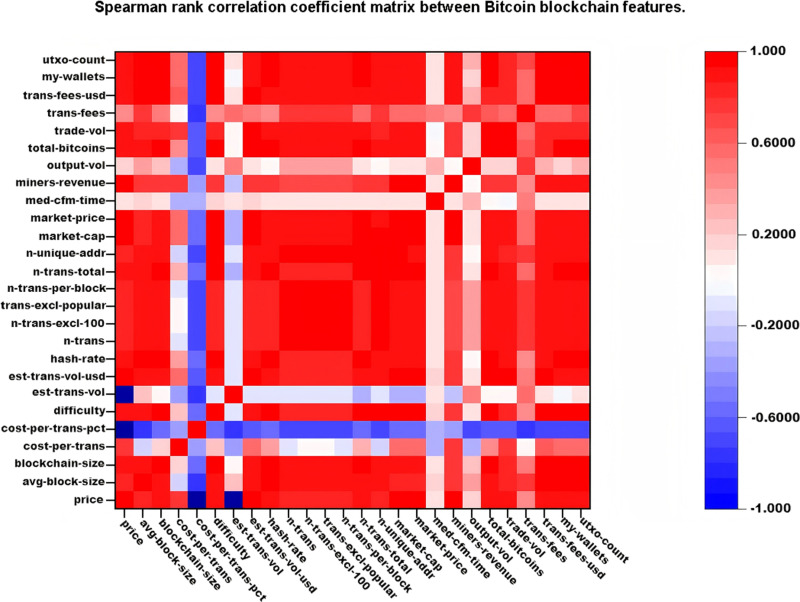
Spearman rank correlation coefficient matrix between bitcoin blockchain features.

To identify the most useful features among those listed in Table 5, a correlation analysis with bitcoin price is performed on the remaining features. Specifically, we calculated Spearman’s rank correlation coefficients and Spearman rank correlation coefficient matrix between bitcoin blockchain features, as shown in Table 6 and Fig 4. Spearman’s rank correlation coefficient is a non-parametric measure used to evaluate the monotonic relationship between two variables. The Spearman correlation between two variables is similar to the Pearson correlation, representing a linear correlation between the ranked values of the two variables. A positive Spearman correlation coefficient between two variables *X* and *Y* indicates that when *X* increases (or decreases), Y also tends to increase (or decrease). Conversely, a negative coefficient indicates that as *X* increases (or decreases), *Y* tends to decrease (or increase). A Spearman correlation of zero indicates no correlation between X and *Y*.

**Table 6:** Spearman correlation coefficients between bitcoin prices and various features.

Bitcoin feature	Coefficient
avg-block-size	0.8479
blockchain-size	0.9017
cost-per-trans	0.7568
cost-per-trans-pct	−0.4734
difficulty	0.8967
est-trans-vol	−0.0565
est-trans-vol-usd	0.9397
hash-rate	0.9021
market-cap	0.9876
market-price	0.9915
med-cfm-time	0.0878
miners-revenue	0.9713
my-wallets	0.8998
n-trans	0.8162
n-trans-excl-100	0.8469
n-trans-excl-popular	0.8233
n-trans-per-block	0.8070
n-trans-total	0.9030
n-unique-addr	0.8655
output-vol	0.1428
total-bitcoins	0.9019
trade-vol	0.8953
trans-fees	0.4297
trans-fees-usd	0.9310
utxo-count	0.9013

**Fig 5:**
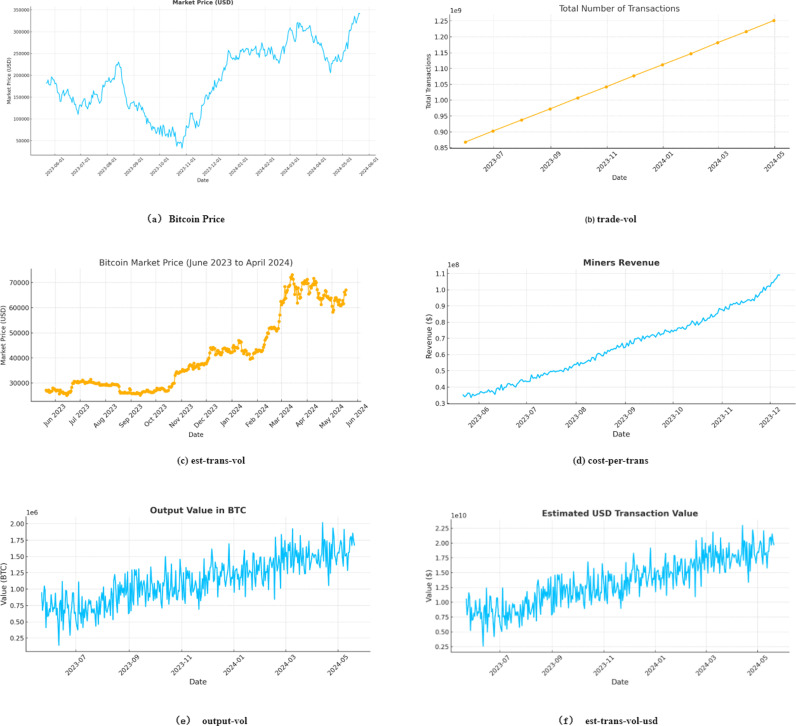
Changes in the values of selected features. (a) Bitcoin price. (b) Trade-vol. (c) Est-trans-vol. (d) Cost-per-trans.

In this study, we excluded features with correlation coefficients less than 0.75, such as cost-per-trans-pct, est-trans-vol, med-cfm-time, output-vol, and transfees, due to their weak relationship with bitcoin price. Features with correlation coefficients greater than 0.95, such as market capitalization, market price, and miner income, are also excluded. In fact, market cap and market price trends are almost identical to bitcoin’s price. Note that market price is the average bitcoin USD market price across major bitcoin exchanges, and market cap is defined as the market price multiplied by the supply of bitcoin in circulation. The trends of some features, which are either highly correlated or not correlated with the bitcoin price, are shown in Fig 5. For example, the trend of trade-vol, shown in Fig 5b with a Spearman correlation of 0.8953, is very similar to that of the bitcoin price shown in Fig 5a. In contrast, the trend of est-trans-vol, shown in Fig 5c with a Spearman correlation of − 0.0565, is not related to the bitcoin price. In total, 18 features, including the bitcoin price, are used to develop prediction models and predict the next bitcoin price. Among the 18 features, the ranges of the values of difficulty, est-trans-vol-usd, hash-rate, my-wallets, trade-vol, and trans-fees-usd are very large. Therefore, the log values of these features are used in the experiment.

## 5. Experiment analysis

### 5.1. Experience and results

All experiments in this article are conducted on a single computer, Win10 64-bit operating system, Intel(R) Core (TM) i7–9750H CPU @2.60 GHz 2.59 GHz, NVIDIA GeForce RTX 2060 GPU and 16GB RAM HP laptop on Run the generated program. Data processing and enhancement via Python. The LSTM model and ARIMA model is implemented using Python3.9 and the Keras deep learning library, and the conformal prediction model is implemented using the Torchcp library. The parameter settings used in the experiments are shown in Table 7.

**Table 7:** The parameter settings used in the experiments.

Module	Parameter settings			
**LSTM**	Layers of LSTM	Hidden size of LSTM	Learning rate	Batch size
	2	64	0.001	16
**CP**	Confidence level		Calibration set size	
	95%		70% Training set 30% Calibration set	

**Fig 6:**
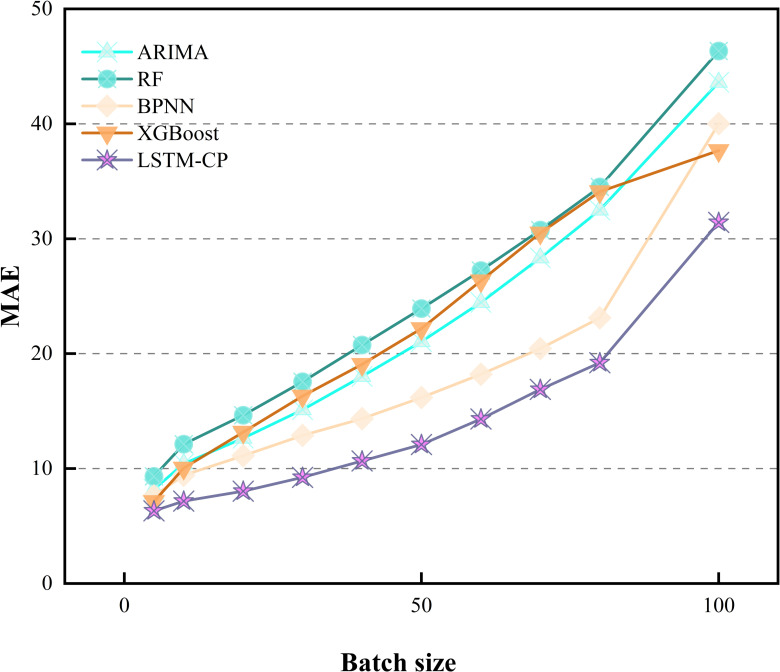
MAE metrics for different models at varying input sizes.

**Fig 7:**
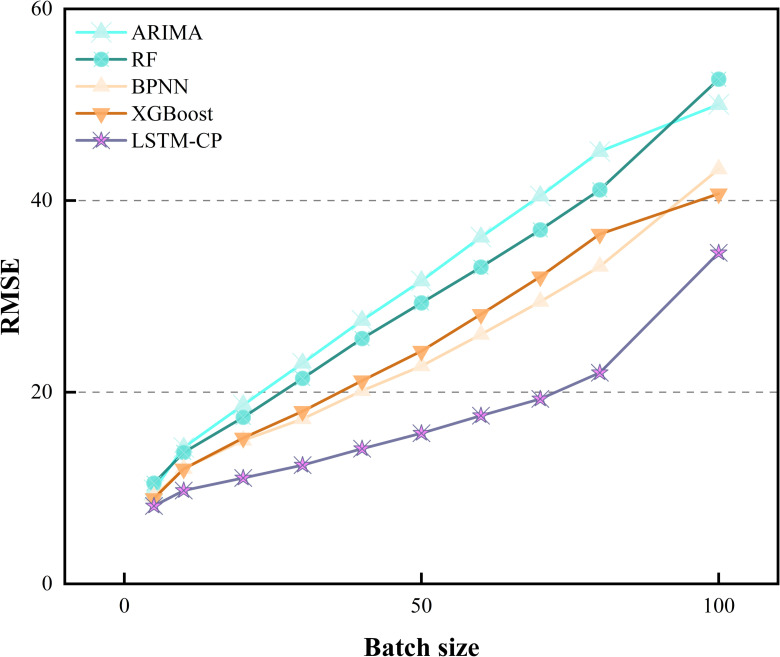
RMSE metrics for different models at varying input sizes.

**Fig 8:**
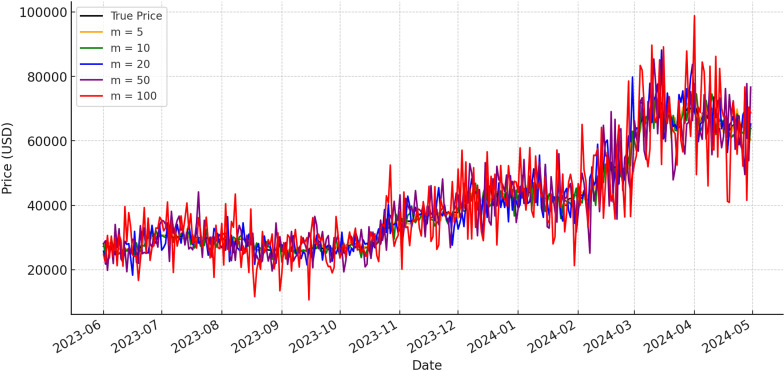
Comparison of predicted values across varying input sequence sizes.

Initially, the input sequence size m is determined through experimental analysis. Table 8 illustrates the influence of m on the regression performance of the LSTM model in relation to MAPE. Optimal values are denoted by underlined numbers for a given window size, while those in bold signify optimal values for a specific prediction model. Notably, all prediction models achieve superior performance when m=5, as lower MAPE values indicate better prediction accuracy. With a smaller input series size, prediction models tend to forecast prices closely aligned with the previous day, thereby minimizing MAPE. However, solely focusing on the changes indicated by the MAPE metric does not fully capture the model’s performance. To provide a more comprehensive evaluation of the model’s performance, Figs 6 and 7 display the MAE and RMSE metrics for different batches. Consequently, selecting a smaller input sequence size is advisable. To provide a more intuitive presentation of the results, the data from 2023-2024 are partially visualized, the results are shown in Fig 8.

**Table 8:** The impact of sequence size *m* on regression (expressed as MAPE, %). Logarithmic values of the primary features, sequential partitioning, and normalization based on the first value are applied.

Size (m)	MAPE
5	**3.68**
10	4.01
20	4.51
50	6.94
100	28.09

**Fig 9:**
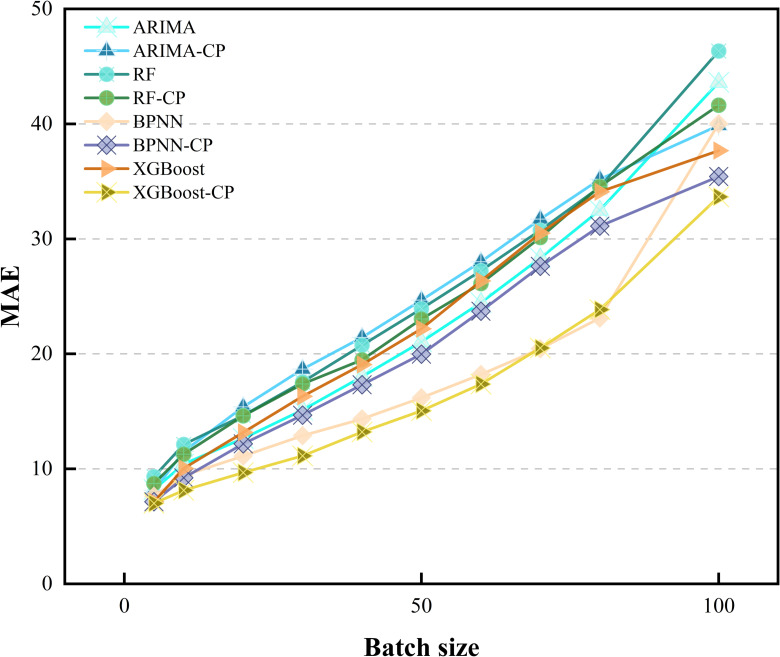
MAE after embedding the CP module into different models.

**Fig 10:**
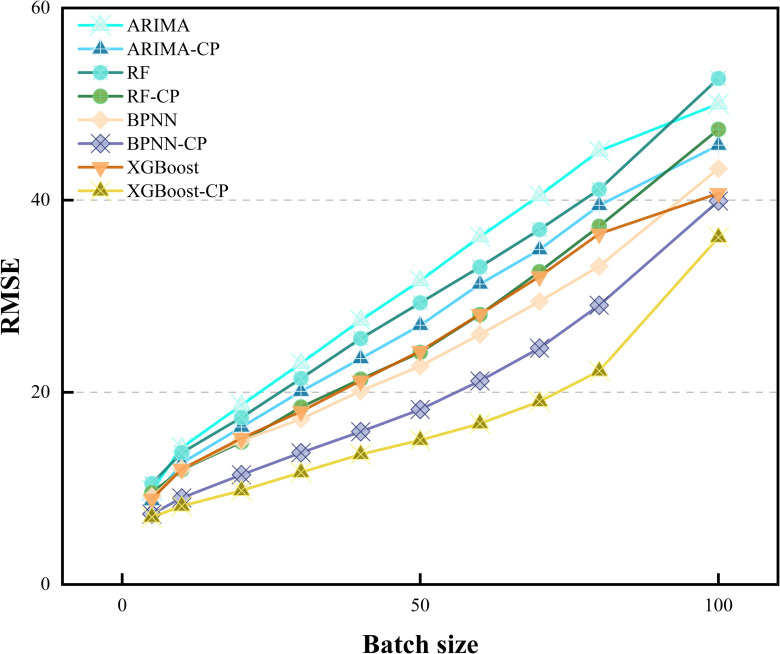
RMSE after embedding the CP module into different models.

However, a natural question arises. To test the generalization of CP, does directly embedding it into existing models also improve their reliability? Therefore, Figs 9 and 10 display the performance of other models after embedding the CP module. Clearly, the performance of all models has improved to varying extents after embedding the CP module.

**Fig 11:**
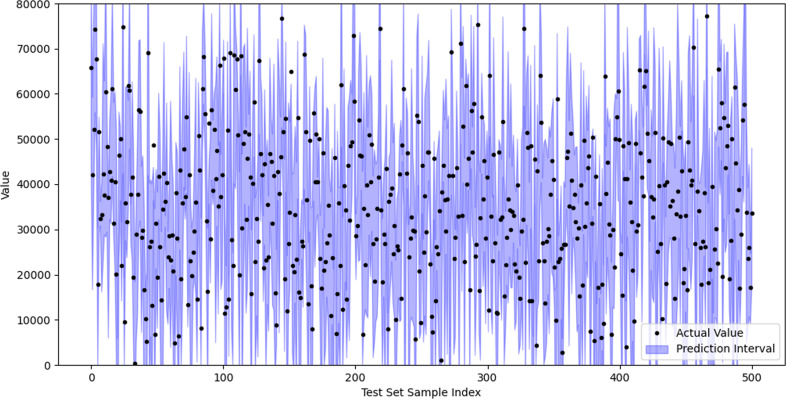
Prediction intervals and actual values.

The results of the conformal prediction model are depicted in Fig 11. Black dots illustrate the actual sample values in the test dataset, which the predictive model aims to forecast accurately. Each blue vertical line represents a prediction interval, signifying the model’s potential prediction range for a sample. Prediction intervals typically capture model uncertainty arising from data noise, imperfections, or limitations in the training sample. The abscissa shows the test dataset sample index, linking each actual value and prediction interval with its corresponding test sample. On the ordinate, actual values (depicted by black dots) represent each sample’s measured value in the test dataset. For the prediction interval (blue vertical line), the ordinate’s upper and lower ends indicate the upper and lower bounds of the prediction, respectively. As shown in Fig 11, approximately 95.2% of the actual values fall within the prediction interval, indicating the model’s prediction interval offers a reasonably accurate range of uncertainty. This underscores our high confidence in the results of the LSTM model.

First, when the prediction interval is too wide, the associated uncertainty is also large, which may hinder effective decision-making. For high-volatility assets, an excessively wide prediction interval may lead to overly cautious risk management strategies and increased exposure to potential risks. For instance, if investors set stop-loss and take-profit points based on the width of the prediction interval, an overly wide interval may result in stop-loss and take-profit points being set too far apart, causing investors to lose control during sharp market fluctuations, potentially amplifying losses. Additionally, rapid market changes require immediate responses, and excessively wide prediction intervals may make decisions seem slow and overly conservative, disregarding the market’s rapid adjustments.

When the prediction interval is too narrow, it indicates that the model’s prediction of future prices is highly accurate, but this may not reflect the true uncertainty. Narrow prediction intervals may lead to overconfidence among decision makers and an underestimation of market risks. In investment decisions, excessive reliance on narrow predictions may prompt overly aggressive actions and the neglect of potential market fluctuations. Narrow predictions may sometimes result from the model’s overfitting of historical data. Overfitting often causes the model to become overly dependent on past market patterns, ignoring potential future changes.

In practice, while a wide range offers a higher confidence level, it may cause investors to miss quick market opportunities or increase risk exposure due to over-conservatism. In this case, there is a significant conflict between the width of the range and the urgency of the decision. Conversely, while a narrow range may improve decision-making accuracy, it may lead investors to overlook the true volatility and potential risks of the market. Particularly in highly volatile markets, a narrow range quickly becomes ineffective.

## 5.2. Discussion

A novel method that combines conformal prediction with LSTM models is proposed in this study, providing multiple improvements for time series forecasting. Specifically, LSTM models address the constraints of other predictive models by capturing the intricate and often non-linear dynamics of bitcoin market behavior, enabling real-time predictions in the volatile bitcoin market environment. LSTM enhances the model’s ability to generalize to unseen data. Moreover, incorporating confidence intervals through conformal prediction adds a probabilistic dimension that enhances interpretability by presenting a range of predicted values. This addition is particularly advantageous for decision-makers across various domains, especially in the intricate realm of bitcoin market forecasting, as it enables the assessment of prediction reliability for informed strategic adjustments. This advancement signifies a crucial stride toward enhancing the decision-making process through meticulous and informed approaches.

Despite its numerous advantages, this approach also faces inevitable limitations. The complexity of this approach demands substantial computational resources and prolonged training periods, posing implementation challenges, particularly when resources are limited or immediate results are desired. Additionally, the accuracy of confidence intervals generated by conformal predictions heavily relies on the representativeness of the calibration dataset. Inadequate reflection of future data distributions in the dataset can severely impact the reliability of confidence intervals, underscoring the significance of high-quality, representative data. Furthermore, noisy data or suboptimal predictions from the LSTM model may lead to overly wide confidence intervals, reducing the precision of confidence measures and emphasizing the need to balance interval width with information value. Currently, there exists a Denoising Diffusion Probabilistic Model (DDPM) [24], which is a class of generative models optimized through a two-step process: injecting noise into the training samples and subsequently removing it. This process consists of a forward phase (diffusion) and a reverse phase (denoising). By training the model to remove the noise introduced during the diffusion process, valid data samples closely aligned with the training data distribution are generated in the inference phase, making it highly suitable for modeling time series patterns and for downstream tasks. Integrating these advanced techniques into the existing LSTM-conformal prediction model is expected to significantly enhance prediction methods in economic scenarios.

In conclusion, while this novel approach is poised to notably enhance the accuracy and reliability of time series forecasting, it also presents challenges necessitating strategic considerations and mitigation efforts to fully leverage its benefits.

## 6. Conclusion

This study presents a novel approach in which LSTM models are integrated with conformal prediction techniques, tailored for bitcoin market prediction. This method enhances the credibility of forecasted values, addressing the primary challenge of accurately predicting market trends. The initial phase of our methodology involves selecting and preprocessing key features extracted from the dataset, including the average block size, total blockchain size, and transaction-related metrics such as average transaction size and transaction cost percentages. Features are selected based on Spearman correlation coefficients, excluding those with values below 0.75 or above 0.95.

The key aspect of the method is the intricate processing of preprocessed data using an LSTM network in sequential time steps. After generating predictions, backpropagation is employed to refine the final output using a loss function and optimizer, ensuring prediction accuracy. Subsequently, preliminary predictions from the LSTM model are incorporated into the conformal prediction model.

In future research, the challenge of managing excessively wide confidence intervals, often caused by significant dataset noise and dependency issues, will be addressed. The forecasting framework will be refined to improve the efficiency and credibility of market sales forecasts, contributing to the knowledge base and advancing predictive analytics in market trend forecasting. Advanced data preprocessing techniques will be explored, LSTM model parameters will be optimized, and other predictive models may be integrated to improve the robustness and accuracy of the methods.
